# Ligation of Intersphincteric Fistula Tract (LIFT) with or Without Injection of Bone Marrow Mononuclear Cells in the Treatment of Trans-sphincteric Anal Fistula: a Randomized Controlled Trial

**DOI:** 10.1007/s11605-022-05316-x

**Published:** 2022-04-25

**Authors:** Mohamed Rezk, Sameh Hany Emile, El Yamani Fouda, Nada Khaled, Mohamed Hamed, Waleed Omar, Wael Khafagy, Ahmed AbdelMawla

**Affiliations:** Colorectal Surgery Unit, General Surgery Department, Mansoura University Hospitals, Mansoura University, 60 El-Gomhouria Street, Mansoura, 35516 Dakahlia Egypt

**Keywords:** LIFT, Bone marrow, Mononuclear cells, Anal fistula, Randomized trial

## Abstract

**Background:**

Ligation of intersphincteric fistula tract (LIFT) is a sphincter-saving procedure used for treatment of complex anal fistula. The current study aimed to assess the outcome of local injection of bone marrow mononuclear cells (BM-MNCs) in conjunction with LIFT as compared to LIFT alone in regards to healing rate, time to healing, and ultimate success rate.

**Methods:**

This was a prospective randomized trial on patients with trans-sphincteric anal fistula. Patients were randomly allocated to one of two equal groups: LIFT and LIFT with BM-MNC injection. The main outcome measures were healing at 10 weeks of follow-up, recurrence after healing, and complications.

**Results:**

Seventy patients (48 male and 22 female) of a mean age of 37.9 ± 10.4 years were included. The mean time to complete healing after LIFT + BM-MNCs was significantly shorter than after LIFT alone (20.5 ± 5.2 vs 28.04 ± 5.8 days; *P* < 0.0001). The ultimate success rates of both groups were similar (LIFT = 60% vs LIFT with BM-MNCs = 68.6%, *P* = 0.62). There was no significant difference in the mean operation time or complication rate between the two groups. Secondary extension and previous anal surgery were significant independent predictors of failure of healing.

**Conclusion:**

LIFT combined with BM-MNC injection was associated with a shorter time to complete healing than LIFT alone. However, BM-MNC injection did not have a significant impact on the overall healing and ultimate success rate.

## Introduction

The main objectives of treatment of anal fistula are drainage of infection, healing of anal fistula, and prevention of recurrence while preserving the anal sphincter function [1]. While simple anal fistulas are commonly treated with fistulotomy, more complex cases necessitate more advanced procedures aiming at preserving the anal sphincters. Seton placement, anal advancement flap, ligation of intersphicnteric fistula tract (LIFT), laser ablation, fistula plug, and video-assisted anal fistula treatment are sphincter-saving procedures used to treat complex anal fistulas [2].

LIFT procedure was first introduced by Rojansakul in 2007, and since then, it has gained popularity due to its promising initial outcomes and technical simplicity [3]. According to meta-analyses, the weighed mean healing rates after LIFT is around 75% over 12 months of follow-up [4]. However, recent studies showed that the success rates of LIFT can be less than 50%. The varying outcomes after LIFT may be attributed to non-standardized enrollment criteria among different studies, variable durations of follow-up, and non-standardized surgical techniques [5].

To improve the outcome of LIFT, some authors used a combined approach of LIFT. Pooled analysis of seven studies including 192 patients revealed a success rate of 83.5% after the combined LIFT approach [6]*.* The use of bone marrow aspirate concentrate (BMAC) in surgery is not entirely new as it has been widely used in the treatment of bone defects [7], mandibular reconstruction [8], maxillary sinus augmentation [9], and in critical limb ischemia [10].

A previous study from our colorectal surgery unit concluded that the use of BMAC to augment external anal sphincter repair strengthens wound healing by transferring cells responsible for healing directly to the site of repair [11]. The current study aimed to assess the outcome of local injection of bone marrow mononuclear cells (BM-MNCs) in conjunction with LIFT as compared to LIFT alone in regard to healing rate, time to healing, and ultimate success rate.

## Patients and Methods

### Study Design and Setting

This was a prospective randomized controlled trial on patients with trans-sphincteric anal fistula. The study was conducted in the Colorectal Surgery Unit Mansoura University Hospital from June 2019 to September 2021. The study was carried out after getting ethics approval from the Institutional Review Board of our institution. Signed informed consent was obtained from every patient before enrollment, highlighting the possible future publication. The trial has been registered in the clinicaltrials.gov under the special identifier NCT05134168 and is reported in line with the Consolidated Standards of Reporting Trials (CONSORT) guidelines.

### Selection Criteria

Adult patients of either gender presented with cryptoglandular trans-sphincteric anal fistula were included. We excluded patients with secondary anal fistula caused by inflammatory bowel disease, malignancy, or irradiation, immunocompromised patients, those with previous pelvic radiotherapy, pregnant women, and patients with ASA (American society of anesthesiologists) III or higher.

### Preoperative Assessment

A detailed history was taken regarding the current complaint and its duration, associated medical conditions, previous surgical operations, constipation, and fecal continence state. The continence status was assessed with the Wexner incontinence score [12]. MRI or endoanal ultrasound (EAUS) (Flex Focus 400 Ultrasound Scanner (BK Medical, Herlev, Denmark) was performed for assessment of the type and branching of the fistula tract and localization of the internal opening.

### Random Sequence Generation

Eligible patients were randomized using randomization software (www.randomization.com) into one of two equal groups: LIFT and LIFT with BM-MNCs. The allocation of patients to each group was concealed using the sealed envelope method. Patients and surgeons were aware of the nature of the trial and group allocations; however, outcome assessors were blinded to the group allocations. Patients could not be blinded to the intervention as they were aware of the aspiration of BM from the iliac spine.

### Procedures

Patients were operated on in the modified lithotomy position under spinal anesthesia. Prophylactic antibiotics in the form of 1 g of cefotaxime and 500 mg metronidazole were given intravenously at the time of the induction to patients in the two groups. The procedures were performed or supervised by three consultants of colorectal surgery with experience in performing the LIFT procedure. None of the patients in the two groups had a drainage seton before surgery. Gentle anal dilatation, insertion of an anal retractor, and EUA were performed in a standardized manner.

#### LIFT Technique

The classical LIFT procedure as described by Rojanasakul et al. [3] was performed. The internal opening was localized by injection of hydrogen peroxide or povidone iodide through the external opening and gently probing the fistula tract. The inter-sphincteric plane was entered via a curvilinear incision. The inter-sphincteric fistula tract was carefully dissected, using scissors and electrocautery.

The intersphincteric tract was raised using a small right-angled clamp. The track was then ligated twice, first close to the internal sphincter then at a distal point with Vicryl 3/0 then the tract was divided between the two ligatures. The remnant of the inter-sphincteric tract or possibly the infected gland was removed. Hydrogen peroxide or povidone iodide was injected through the external opening once more to confirm that the track was completely divided and sealed. The external opening was thoroughly curetted with a metallic curate and was then adequately drained. The last step was the closure of the inter-sphincteric incision loosely with interrupted Vicryl 3/0.

#### LIFT and BM-MNCs Injection

**Fig. 1:**
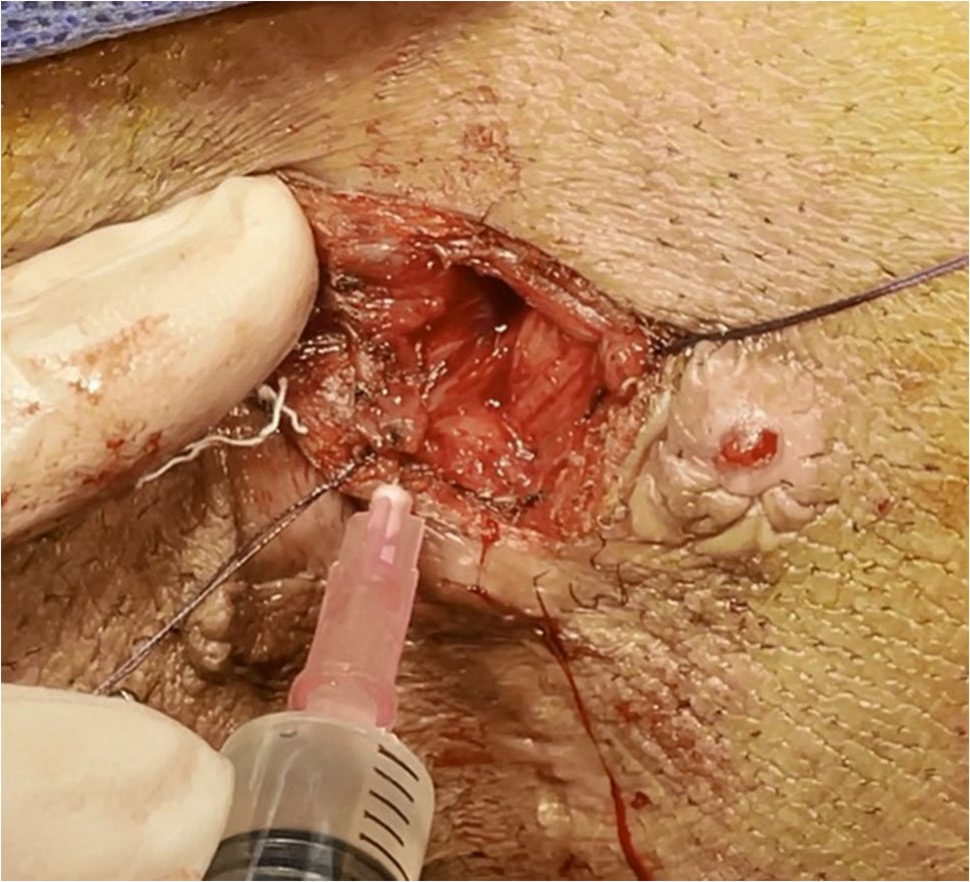
Injection of bone marrow mononuclear cells around the ligated fistula tract

**Fig. 2:**
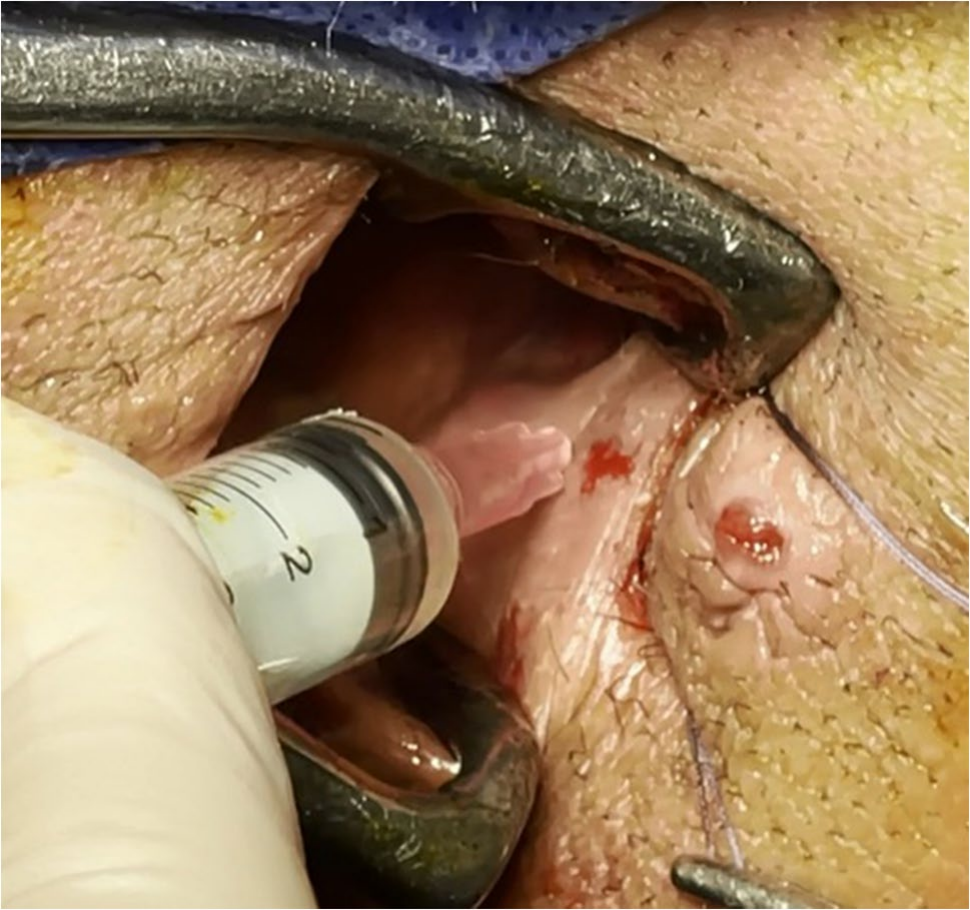
Injection of bone marrow mononuclear cells around the internal opening

LIFT was done according to the abovementioned steps, followed by injection of 2 mL of BM-MNCs using two syringes (29 gauge) along the ligated fistula tract in the intersphincteric space (Fig. 1) and the internal opening (Fig. 2). The injection of BM aspirate ensured that it was retained inside the tissue and did not escape through sutures.

#### Preparation of the BM-MNCs

One hour before surgery, patients were placed in the left lateral position and the lower part of the back was prepped by povidone-iodine (10%) and draped. A small stab was made in the skin over the posterior superior iliac spine (PSIS) under local anesthesia and a special trocar and cannula (Jamshidi needle) were introduced into the bone marrow cavity of the iliac bone. Ten-milliliter bone marrow was aspirated in a syringe pre-flushed with heparin (1000 units/mL). The stab wound was dressed, and the bone marrow syringe was shacked gently for 5 min; then, it was taken to the laboratory for preparation of BMAC.

The MNCs were isolated from the bone marrow using density gradient centrifugation (DGC). Five-milliliter bone marrow were diluted by 5 mL phosphate buffer saline (PBS) then added to a 5-mL Falcon tube containing 5 mL Lymphoflot (BioRAD Company, Germany). Then, the tube was centrifuged at 4000 rpm (round per minute) for 20 min; after centrifugation, the tube contents were separated into 4 layers: plasma on the top, a thin layer of MNCs, Lymphoflot, and then the remaining components of bone marrow (red in color) at the bottom. MNCs were aspirated and washed three times using PBS (they were put in a new tube then PBS was added, and the tube was centrifuged at 1000 rpm for 10 min after which the old PBS was removed and new PBS was added, and the process was repeated). In the end, MNCs were re-suspended in 2 mL PBS and were sent to the operation room.

### Post-operative Care

Patients in the two groups were discharged on the next day of surgery and were prescribed oral quinolone antibiotics for 3 days and stool softeners for 1 week. Instructions on wound dressing using a Sitz bath every 6 h were given to the patients.

### Follow-up

Patients were followed up at the outpatient clinic at 1 and 2 weeks, 1, 3, and 6 months postoperatively. At each visit, patients were assessed clinically for fistula healing, continence state using Wexner incontinence score, and postoperative complications including infection, bleeding, hematoma, and incontinence. Assessments were made by a surgical resident who was not aware of group allocations and by one of the study authors who did not influence the outcome of assessments.

### Study Outcomes

The primary outcome was complete fistula healing at 6 months. The secondary outcomes were operation time, length of hospital stay, continence change, and complications. The operation time was measured from the onset of the incision until closure of the intersphincteric wound. The time taken to obtain and prepare the BM-MNCs was not included in the operation time.

Fistula healing was defined as the closure of the internal and external openings without any discharge. Failure of healing was defined if complete closure of the external opening did not occur at 10 weeks after surgery. Recurrence was defined as reappearance of the external opening after complete healing, appearance of new external opening, and recurrence of symptoms after complete resolution on follow-up. Ultimate success was defined as complete fistula healing 6 months after surgery with the absence of recurrence.

### Sample Size Calculation

The sample size was calculated using sample size and power software ((http://clincalc.com/stats/samplesize.aspx) based on the primary endpoint of the study (complete healing at 6 months of follow-up). In light of previous literature on LIFT [4, 13–16], the success rate of LIFT ranged between 47 and 79% with a mean success rate of 60%; we assumed that injection of BM-MNC may increase the success rate by an additional 30%; therefore, a minimum of 62 patients, equally divided on both groups, was required to obtain study power of 80% with alpha set at 5%. To compensate for drop-out and loss to follow-up, 70 patients were initially included in the trial.

### Statistical Analysis

Data were analyzed using Excel and SPSS (Statistical Package for Social Science) version 23 programs under Microsoft Windows. Quantitative data were expressed as mean and SD or median and range according to data normality. Student *t*-test was used to analyze quantitative data whereas chi-square or Fisher’s exact test was used for categorical data. An intention to treat analysis and per-protocol analysis were performed. Multivariate binary logistic regression analysis of the predictors of failure of healing was conducted. The area under the curve (AUC) of the model used was calculated to determine its discriminatory power. A per-protocol analysis of the study outcomes was used. A *P*-value less than < 0.05 was considered significant.

## Results

### Patients’ Characteristics

**Fig. 3:**
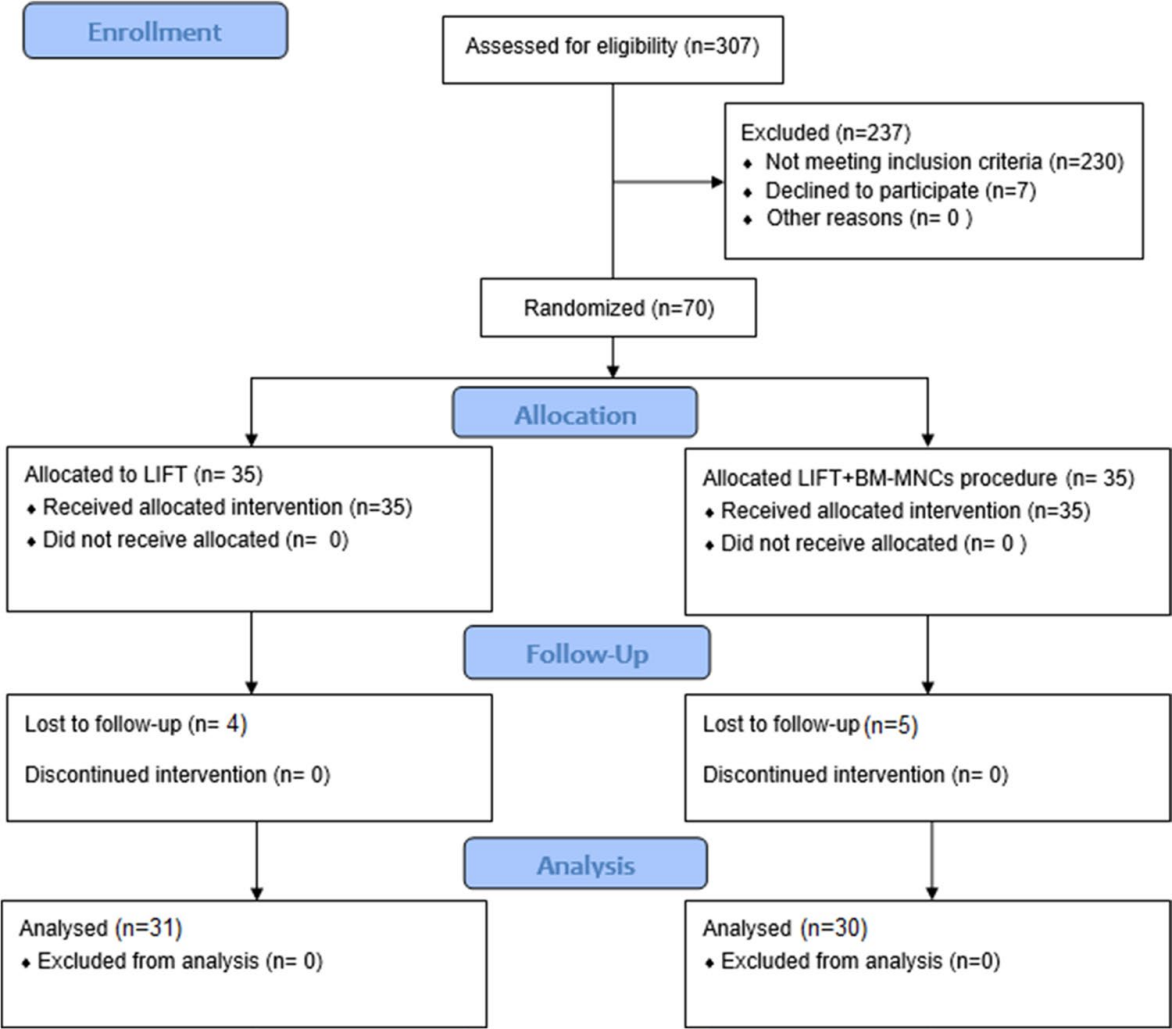
CONSORT flow chart illustrating the process of patient recruitment and selection

During the period of the study, 307 patients with anal fistula were assessed; 237 patients did not fulfill the inclusion criteria and were excluded; the CONSORT flow chart is shown in Fig. 3. Thus, the present study included a total of 70 patients. Patients were 48 (68.7%) male and 22 (31.3%) female. The mean age of patients was 37.9 ± 10.4 years and the mean BMI was 26.7 ± 12.3 kg/m^2^. Seven (10%) patients had type 2 DM and 14 (20%) were smokers.

Sixteen (22.8%) patients had previous anal surgery, of whom 12 had previous fistula surgery. Thirty-five patients were randomized to have LIFT, and 35 had LIFT plus BM-MNC injection. There were no significant differences between the two groups in regards to baseline patient characteristics including age, sex distribution, BMI, and previous anal surgery as shown in Table 1.

**Table 1:** Baseline patient characteristics in the two groups

Variable	LIFT	LIFT + BMMN	*P* value
Number	35	35	**––-**
Mean age in years	39.9 ± 9.2	35.9 ± 11.2	0.1
Males (%)	23 (65.7)	25 (71.4)	0.79
Mean body mass index in kg/m^2^	27.05 ± 2.5	26.4 ± 2.6	0.29
Smoking (%)	6 (17)	8 (22.8)	0.76
Diabetes mellitus (%)	3 (8.5)	4 (11.4)	0.99
Previous anal surgery (%)	6 (17)	10 (28.5)	0.39

^*^LIFT, ligation of intersphincteric fistula tract

^*^BM-MNCs, bone marrow mononuclear cells

### Fistula Characteristics

Overall, there were 22 (31.4%) anterior fistulas, 19 (27.1%) posterior fistulas, and 20 (28.5%) lateral fistulas. Nine (12.8%) patients had a secondary extension of the primary tract and/or horseshoe fistula. Nine (12.8%) patients had multiple external openings of the fistula. Twenty-seven patients had a history of perianal abscess drainage. There were no significant differences between the two groups in regard to fistula characteristics as shown in Table 2.

**Table 2:** Fistula characteristics in the two groups

Variable	LIFT (*n* = 35)	LIFT + BMMN (*n* = 35)	*P* value
Fistula position (%)			
Anterior Posterior Lateral	10 (28.5) 12 (34.3) 9 (25.7)	12 (34.3) 7 (20) 11 (31.4)	0.43
Secondary extension (%)	2 (5.7)	4 (11.4)	0.67
Horse-shoe (%)	2 (5.7)	1 (2.8)	0.9
Multiple openings (%)	4 (11.4)	5 (14.3)	0.99
Previous fistula surgery (%)	5 (14.3)	7 (20)	0.75
Previous abscess drainage (%)	16 (45.7)	11 (31.4)	0.32

^*^LIFT, ligation of intersphincteric fistula tract

^*^BM-MNCs, bone marrow mononuclear cells

### Primary Outcome

Overall, 31 (88.5%) patients in the LIFT group and 30 (85.7%) in the LIFT + BM-MNCs completed 6 months of follow-up whereas nine patients were lost to follow-up. Among the 31 patients in the LIFT group, 10 patients did not achieve healing versus 8 out of 30 patients in the LIFT + BM-MNC. For patients who achieved complete healing, recurrence of the fistula was recorded in 4/21 (19%) patients in the LIFT group versus 3/22 (13.6%) patients in the LIFT + BM-MNCs group (*P* = 0.69).

As per protocol analysis, ultimate success at 6 months was achieved in 17/31 (54.8%) in the LIFT group versus 19/30 (63.3%) in the LIFT + BM-MNCs group (*P* = 0.68). As per intention to treat analysis, ultimate success at 6 months was achieved in 21/35 (60%) after LIFT versus 24/35 (68.6%) after LIFT with BM-MNCs (*P* = 0.62) (Table 3).

**Table 3:** Healing in the two group

Variable	LIFT (*n* = 35)	LIFT + BMMN (*n* = 35)	*P* value
Mean healing time in days	28.04 ± 5.8	20.5 ± 5.2	** < 0.0001**
Healing at 4 weeks (%)	15 (42.8)	25 (71.4)	**0.03**
Healing at 6 weeks (%)	24 (68.6)	27 (77.1)	0.59
Healing at 10 weeks (%)	25 (71.4)	27 (77.1)	0.78
Ultimate success at 6 months (%)	21 (60)	24 (68.57)	0.62
Mean follow-up in months	8.5 ± 2.8	7.3 ± 2.75	0.07

^*^LIFT, ligation of intersphincteric fistula tract

^*^BM-MNCs, bone marrow mononuclear cells

The mean time to complete healing after LIFT + BM-MNCs was significantly shorter than after LIFT alone (20.5 ± 5.2 vs 28.04 ± 5.8 days; *P* < 0.0001).

Patients with failure of primary healing (*n* = 18) and recurrence after healing (*n* = 7) were managed with fistulotomy for inter-sphincteric fistula (*n* = 7) or seton placement (*n* = 4) for trans-sphincteric fistula. Fourteen patients, all who had failed primary healing, chose to pursue medical care in other hospitals.

### Secondary Outcomes

There was no significant difference in the mean operation time between the two groups (26.02 vs 26.7 min, *P* = 0.7). Two complications were recorded in the group whereas no complications were recorded in the LIFT group (*P* = 0.49). Two patients developed an inter-sphincteric collection at 4–5 days after LIFT + BM-MNCs which was managed conservatively by antibiotics and simple drainage and dressing. No affection of the continence state was observed in any of the patients in the two groups with a median postoperative Wexner incontinence score of zero (Table 4). Patients in the BM-MNC group did not have any adverse effects of BM aspiration, except for mild pain at the aspiration site that was relieved with paracetamol.

**Table 4:** Operation time and complications in the two group

Variable	LIFT (*n* = 35)	LIFT + BMMN (*n* = 35)	*P* value
Mean operation time in minutes	26.02 ± 6.9	26.7 ± 8.03	0.7
Complications (%)	0	2 (5.7)	0.49
Incontinence (%)	0	0	0.99
Median Wexner score	0	0	––-

^*^LIFT, ligation of intersphincteric fistula tract

^*^BM-MNCs, bone marrow mononuclear cells

### Factors Associated with Failure

As shown in Table 5, secondary extension of the primary tract, horse-shoe fistula, and previous fistula surgery was significantly associated with failure of healing of anal fistula. Age, sex, BMI, DM, smoking, fistula location, and type of procedure were not associated with failure.

**Table 5:** Univariate analysis of factors associated with failure of healing

Variable	Failure (*n* = 25)	Success (*n* = 45)	*P* value
Mean age in years	35.6 ± 9.6	39.2 ± 10.7	0.17
Male (%)	17 (68)	31 (68.9)	0.94
Mean BMI in kg/m2	26.1 ± 2.7	27.1 ± 2.5	0.12
DM (%)	4 (16)	3 (6.7)	0.24
Smoking (%)	7 (28)	7 (15.5)	0.35
Fistula location (%)			
Anterior Posterior Lateral Multiple	8 (32) 5 (20) 6 (24) 6 (24)	14 (31.1) 14 (31.1) 14 (31.1) 3 (6.7)	0.19
Secondary extension (%)	5 (20)	1 (2.2)	**0.02**
Horse-shoe fistula (%)	3 (12)	0	**0.04**
Previous fistula surgery (%)	9 (36)	3 (6.7)	**0.005**
Previous abscess drainage (%)	12 (48)	15 (33.3)	0.34
Procedure			
LIFT LIFT + BM-MNCs	14 (56) 11 (44)	21 (46.7) 24 (53.3)	0.62

^*^LIFT, ligation of intersphincteric fistula tract

^*^BM-MNCs, bone marrow mononuclear cells

^*^DM, diabetes mellitus

The significant factors associated with failure of healing revealed by the univariate analysis were entered into multivariate analysis. Secondary extension (*OR* = 12.8, 95% *CI*: 1.2–133.2, *P* = 0.03) and previous anal surgery (*OR* = 11.1, 95% *CI*: 2.5–49.2, *P* = 0.002) were significant independent predictors of failure of healing whereas horse-shoe fistula did not attain statistical significance (*P* = 0.99).

The AUC of the model was 0.763 (95% *CI*: 0.634–0.892) with a standard error of 0.06. Hosmer–Lemeshow test had an insignificant *P*-value of 0.98, implying a good fit of data to the model used.

## Discussion

To improve the outcome of LIFT, it has been combined with adjunct steps. Popular combined techniques include the LIFT plus procedure, bio-LIFT, and LIFT plug. LIFT plus entails classical LIFT followed by coring out of the external portion of the fistula tract [17] whereas bio-LIFT and LIFT plug entail placement of a bio-mesh or a plugin in the intersphincteric space or the external tract to reinforce the closure of the fistula tract [18, 19]. A further modification of the LIFT procedure used a human acellular dermal matrix as a bioprosthetic plug with an overall success rate of 95% and a median healing time of 4 weeks [20].

Another concept devised to improve the outcome of LIFT was to inject certain materials in the inter-sphincteric space to hasten and enhance healing. Madbouly et al. [21] randomized 98 patients with trans-sphincteric fistulas to LIFT with injection of platelet-rich plasma (PRP) in the inter-sphincteric space and LIFT only. Patients who underwent LIFT-PRP had a significantly shorter time to healing and a higher ultimate success rate than the control group (85.7% vs 65.3%).

In line with the previous concept, the current study tried to improve the outcome of LIFT by injecting BM-MNCs at both the internal opening and along the tract in the intersphincteric space. The MNC fraction represents 85% of the composition of the BMAC [22] and contains the cells responsible for wound healing, which are the fibrocyte progenitors and the endothelial progenitor cells. These cells migrate to wound sites and differentiate into fibroblasts that deposit collagen and extracellular matrix, and endothelial cells that form new blood vessels, resulting ultimately in the formation of granulation tissue which matures into a fibrous scar [23].

The present study included 70 patients with trans-sphincteric fistula which is the most common type of complex anal fistula. Patients were mostly male of middle age which is in concordance with the literature on anal fistula, including a gender-based analysis of the outcome of anal fistula surgery by our unit [24]. Both groups had comparable demographics and fistula characteristics which can reflect minimal selection bias owing to adequate randomization of patients to each group.

The primary outcome for this study was the healing of the anal fistula after LIFT. Healing was assessed in terms of the rate of complete healing and also the time needed to achieve healing. Although the injection of BM-MNCs achieved a higher success rate than LIFT alone, this result was not statistically significant, perhaps due to the small numbers included in each group. The present study was powered on the assumption that the injection of BM aspirate would improve healing after LIFT by 30%. Although the ultimate healing after LIFT combined with BM aspirate was higher than LIFT alone, the difference in healing rates did not reach the assumed difference of 30% and thus the *P*-value was not significant. Perhaps, if future trials used a different assumption of a smaller improvement (e.g., 15%), a significant difference in healing between the two groups may be obtained, yet larger numbers will be required.

On another hand, BM-MNC injection served to accelerate healing after LIFT significantly owing to the healing-promoting properties of BM-MNCs as aforementioned. The mean duration to complete healing after LIFT combined with BM-MNC injection was 8 days shorter than healing after LIFT alone. Overall, the ultimate success rates of both groups were similar, and this finding may imply that the injection of BM-MNCs, although accelerated healing, did not improve the chance of healing. This observation might be related to the factors governing the success of the LIFT procedure. It has been established that LIFT may not be a suitable procedure for all types of anal fistula. That is why careful selection of patients and tailoring of treatment to each patient is vital.

The current literature has identified some factors associated with failure of healing after LIFT. One meta-analysis found that horseshoe fistula, associated Crohn’s disease, and a history of previous fistula surgery were significantly associated with failure after LIFT [4]. Another study reported that some technical factors may also contribute to failure after LIFT. These factors included inadequate drainage of the intersphicnetric space, residual necrosis, and infected tissues in the inter-sphincteric groove; injury of the internal anal sphincter and anal canal mucosa; and incomplete ligation of the fistula tract [25].

In line with the existing literature, our analysis revealed that secondary extension of the primary tract, horse-shoe fistula, and previous fistula surgery were significantly associated with failure of healing of anal fistula after LIFT. Therefore, although BM-MNCs may enhance healing of anal fistula when combined with LIFT, its effect may be limited if one or more of these predictive factors of failure are present.

Madbouly et al. [21] injected PRP in conjunction with LIFT which has a similar concept to our study. The authors also reported accelerated healing with the injection of PRP, yet the ultimate success rate was significantly higher with PRP, unlike the present study. This may be attributed to the larger numbers and longer follow-up in their study as compared to the present study.

In absence of predictors of failure, the injection of BM-MNCs may hasten healing. Indeed, at 4 weeks of follow-up, 71.4% of the LIFT-BM-MNCs group achieved complete healing versus around 43% of patients in the LIFT group. This accelerated healing is mostly explained by the biologic properties and impact of BMMN on healing as aforementioned.

The clinical implication of our findings is that the accelerated healing induced by BM-MNCs may impact the patients’ recovery and quality of life. Patients who had expedited healing may return to work and daily activities faster than patients with delayed healing. In addition, accelerated healing obviate the need for daily dressing and Sitz baths which in consequence minimizes patient inconvenience and treatment-related costs.

The limitations of the present study include being a single-center study, the open-label nature of the trial, small numbers included, and short follow-up. However, being the first report on the effect of BMMN injection on the outcome of LIFT, the present study provides useful insights about the utility and role of this kind of therapy which needs to be further studied in larger, multicenter studies with longer follow-up.

## Conclusion

LIFT combined with BM-MNC injection was associated with a shorter time to complete healing than LIFT alone. However, BM-MNC injection did not have a significant impact on the overall healing and ultimate success rate. Secondary extension, horse-shoe fistula, and previous fistula surgery are associated with higher failure rates after LIFT; thus, other procedures may be better selected for these patients.
